# Quantifying Inter-Ply Friction and Clamping Effects via an Experimental–Numerical Framework: Advancing Non-Coherent Deformation Control of Uncured Metal–Fiber-Reinforced Polymer Laminates

**DOI:** 10.3390/polym17172330

**Published:** 2025-08-28

**Authors:** Yunlong Chen, Shichen Liu

**Affiliations:** Department of Aerospace Vehicle Design Engineering, School of Aerospace Engineering, Xiamen University, Xiamen 361105, China

**Keywords:** metal-fiber reinforced polymers, inter-ply friction characterization, process optimization, numerical simulation, non-coherent deformation

## Abstract

Pre-stacked uncured metal–fiber-reinforced polymer (FRP) laminates, which are critical for aerospace components like double-curved fuselage panels, wing ribs, and engine nacelles, exhibit better deformation behavior than their fully cured counterparts. However, accurate process simulation requires precise material characterization and process optimization to achieve a defect-free structural design. This study focuses on two core material behaviors of uncured laminates—inter-ply friction at metal–prepreg interfaces and out-of-plane bending—and optimizes process parameters for their non-coherent deformation. Experimental tests included double-lap sliding tests (to quantify inter-ply friction) and clamped-beam bending tests (to characterize out-of-plane bending); a double-curved dome part was designed to assess the effects of the material constituent, fiber orientation, inter-ply friction, and clamping force, with validation via finite element modeling (FEM) in Abaqus software. The results indicate that the static–kinetic friction model effectively predicts inter-ply friction behavior, with numerical friction coefficient–displacement trends closely matching experimental data. Additionally, the flexural bending model showed that greater plastic deformation in metal layers increased bending force while reducing post-unloading spring-back depth. Furthermore, for non-coherent deformation, higher clamping forces improve FRP prepreg deformation and mitigate buckling, but excessive plastic deformation raises metal cracking risk. This work helps establish a combined experimental–numerical framework for the defect prediction and process optimization of complex lightweight components, which address the core needs of modern aerospace manufacturing.

## 1. Introduction

**Figure 1:**
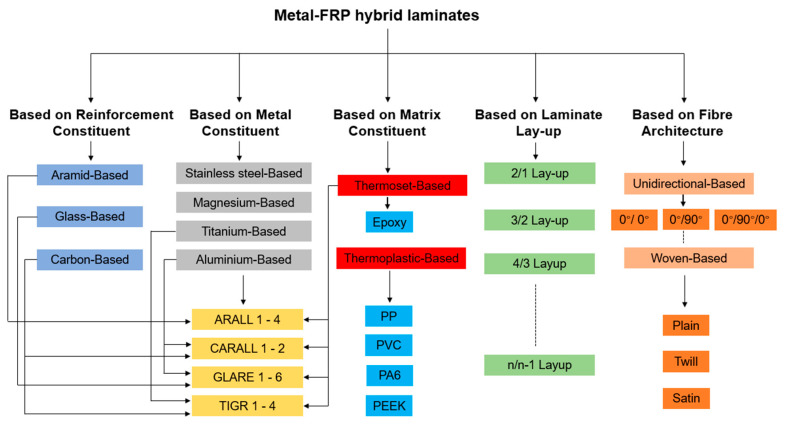
Typical classification of metal–FRP hybrid laminates.

In aerospace manufacturing, reducing fuel consumption and improving structural reliability drives demand for lightweight, high-strength metal–fiber-reinforced polymer (FRP) laminates which are widely used in critical components like curved fuselage panels and high-load wing ribs [1,2]. This hybrid combination creates a material which has significant weight reduction, excellent specific strength, higher stiffness, and more fatigue resistance than the monolithic metal sheet, as well as better impact strength and damage tolerance compared with full-polymer composites [3,4]. A wide range of combinations are available according to the type of metal sheet, the choice of fiber and resin, the number and thickness of layers, etc. [5,6]. The metal alloy sheets currently being used are aluminum, titanium, stainless steel, or magnesium, and fiber-reinforced polymers (FRPs) can be made from dry fibers such as aramid, carbon, or glass fibers with the infusion of resin or prepregs made by pre-impregnated fibers and a partially cured polymer matrix [7,8]. In addition to the traditional classification of metal–FRP laminates, the constituent of composite layers can also consist of woven fabrics and thermoplastic resins instead of unidirectional (UD) reinforcements and thermoset epoxies [9,10]. The typical classification of metal–FRP hybrid laminates based on the material constituents, laminate layups, and fiber architectures is presented in Figure 1.

**Figure 2:**
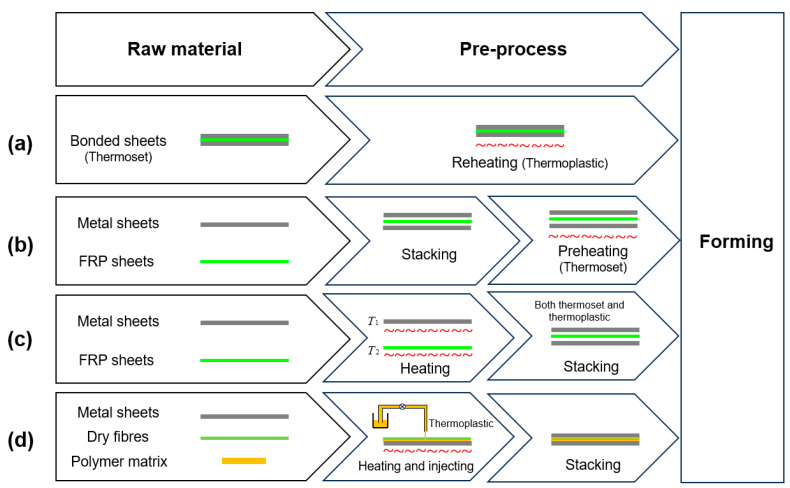
Processing strategies for the forming of metal–FRP laminates: (**a**) Strategy one; (**b**) Strategy two; (**c**) Strategy three; (**d**) Strategy four.

The most common and simple method for forming metal–FRP laminates is layup, followed by autoclave curing. This method suits large components such as fuselage and wing panels, and yields products with good forming quality and mechanical properties, but its applicable geometries are greatly limited by curvature [5,11]. As the metal sheets are not deformed plastically at these processing temperatures, the waviness generated at the edges during layup can be suppressed at a high curing pressure, even though the residual stresses are remained in the panels. Other forming methods for large components by metal–FRP laminates such as press brake bending [12], stretch forming [13], shot peening, or incremental sheet forming [14] and laser forming [15] are described in the literature, including some benefits and drawbacks. For the forming of small- and medium-sized components with relatively small radii and complex shapes, the concept of press forming is introduced, whereby the sheets are pressed using a die and shaped by the deforming force. This concept has proven to be a well-developed mass production technology for forming metal sheet components and also FRP components in recent years [16,17]. Therefore, the method of press forming can be utilized to form metal–FRP laminates for the hybrid material with a thermoplastic matrix because of the short processing time and the improved formability of FRP layers at elevated temperatures. However, the higher processing temperature, which may affect the heat treatments of the metal sheet and induce higher residual stresses, hinders its development [18]. The methods can be categorized into four processing strategies with various raw materials and pre-process steps before forming, as schematically illustrated in Figure 2.

**Figure 3:**
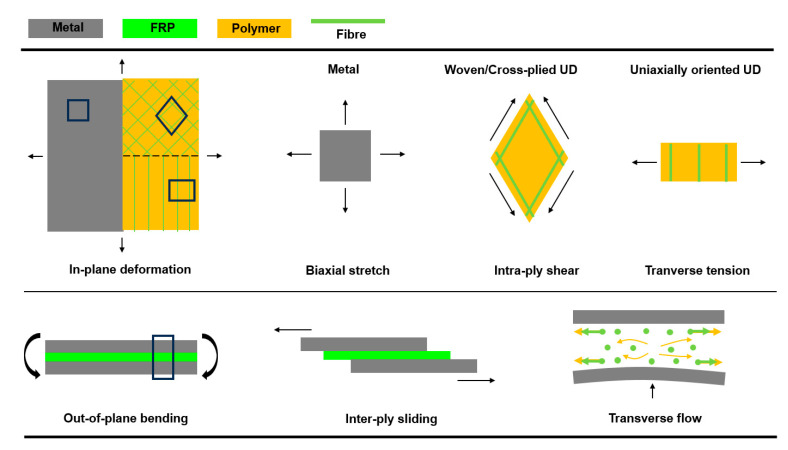
Primary deformation mechanisms for metal–FRP laminates during forming.

During the forming process of metal–FRP laminates, the material properties and layer interactions are the key indexes for evaluating the deformability of the hybrid materials. The primary deformation mechanisms, shown in Figure 3, reveal that the forming behavior of metal–FRP laminates is determined by the deformation mechanisms of metal and FRP sheets, as well as the movements between various material constituents. Regarding the deformation modes involved in sheet metal forming, the in-plane deformation of biaxial stretch is dominated for the forming of three-dimensional (3D) shapes. In addition, the mechanical responses of metal sheets can be either obtained from the uniaxial tensile tests [19] or characterized through the forming limit diagram (FLD) in different test methods [20,21,22]. The deformation mode for FRP varies with different types of fiber reinforcements, including woven and unidirectional (UD), with UD being classified as cross-plied UD and uniaxially oriented UD. The dominating deformation mechanism for woven fabrics or cross-plied UD fibers in FRP sheets is intra-ply shear, which represents the rotation of nearly inextensible fibers. The intra-ply shear mechanism, also named the Trellis effect, is mainly responsible for allowing the FRP sheet to be formed into three-dimensional (3D) shapes [23,24]. Transverse tension is regarded as the main deformation mechanism in uniaxially oriented UD polymers during forming, as the tension force along the transverse direction is much lower than the shear force. Additionally, the effect of intra-ply tension along the longitudinal direction for the FRP sheets can be neglected due to the limited failure strain of the UD fibers [25,26].

The movements between metal sheets and FRP sheets should also be noted as significant factors affecting laminate deformability. Out-of-plane bending is one of the deformation mechanisms in forming metal–FRP laminates, and the bending performance depends on the type of metal and FRP material, as well as the inter-ply interaction of sliding and through-thickness behavior of transverse flow. The bending behavior for sheet metal forming has been well illustrated in the literature [27,28], but the bending of an FRP sheet seems to be less important due to the fact that the bending resistance is usually orders of magnitude lower than the intra-ply shear resistance for uncured polymers [29,30]. Inter-ply sliding is also one of the deformation mechanisms for the bending of metal–FRP laminates, especially for the forming of single-curved components. Notably, existing studies on inter-ply friction of metal–FRP laminates (FMLs) predominantly focus on fully cured or semi-cured systems, which fails to address the unique friction characteristics of uncured prepregs, i.e., a critical gap, as the pre-curing forming stage directly determines the occurrence of defects like wrinkles or cracks. For instance, Zal et al. [31] and Liu et al. [32] investigated inter-ply sliding in FML forming, but relied on simplified friction models (e.g., constant-coefficient penalty friction) or cured-laminate data, which cannot capture the dynamic friction behavior of uncured systems (where resin viscosity, interfacial asperity contact, and temperature jointly regulate friction). Rashidi et al. [33] characterized the inter-ply shear of prepreg fabrics but focused on dry or semi-cured states, without establishing a quantitative friction model for metal–prepreg interfaces in uncured FMLs. In contrast, this study develops a static–kinetic friction model specifically for uncured metal–prepreg interfaces, quantified via a custom double-lap sliding test that systematically varies sliding rates, temperatures, and normal forces. This model, validated by matching numerical and experimental friction coefficient–displacement trends, uniquely captures the transient transition from static to kinetic friction, an ability lacking in earlier constant-coefficient- or brittle-failure-oriented cohesive zone models [34].

**Figure 4:**
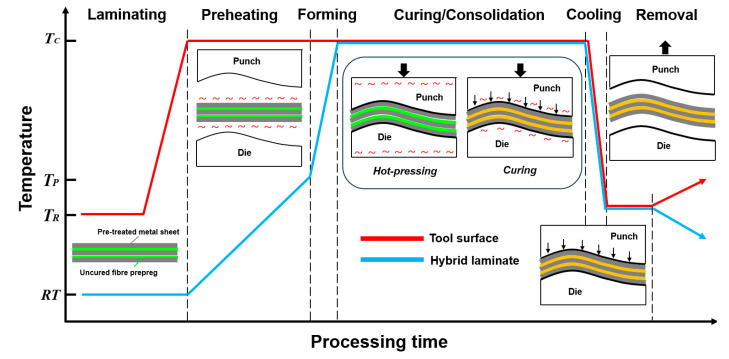
Processing temperature–time profile of the tool surface and hybrid laminate in a processing cycle [37,38].

The forming process of metal–FRP laminates is difficult, especially for epoxy-based hybrid materials, due to the requirements for various forming and curing stages, as well as complex deformation mechanisms. In order to improve the formability of such hybrid laminates, a hot pressing cycle is proposed and the processing temperature-time profile in the cycle is shown in Figure 4. The hot pressing cycle involves a laminate preparing and preheating process, formed of uncured laminate, consolidation, or (partial) curing in a same mold, as well as the cooling and removal of the component. This method contributes to better formability of metal–FRP laminates based on the understanding of deformation mechanisms for both material layers and the optimization of processing parameters in a hot pressing cycle [34,35]. For example, the metal sheets are usually deformed into three-dimensional shapes by bending and in-plane plastic deformation, while the deformation of a fiber-reinforced prepreg is always achieved by inter-ply sliding in-between the layers and intra-ply shear within the layers. All the mechanisms need to be combined and considered in the proposed hot pressing cycle. In addition, the processing parameters, such as preheating temperature and time, curing pressure and time, etc., should also be studied and optimized for the processing of uncured hybrid laminates. Then, the clamping force, a key parameter for coordinating inter-layer deformation in uncured FMLs, has been treated as a secondary or fixed variable in earlier research. Harrison et al. [18] and Ding et al. [11] discussed the press forming of FMLs, but only tested single-clamping pressures (or zero clamping), omitting analysis of how clamping interacts with flexural bending, spring-back, or inter-ply friction. Erland et al. [36] noted intra-ply shear in uncured prepregs, but ignored the role of clamping in restricting material flow and mitigating prepreg buckling. To fill this gap, the present study systematically investigates clamping effects via a custom clamped-beam bending test. Here, how clamping pressure enhances bending resistance and reduces spring-back depth for uncured metal–FRP laminates, never investigated in prior work, was quantified. Furthermore, the clamping force coupled with inter-ply friction and fiber orientation was studied to analyze their combined impact on non-coherent deformation in 3D double-curved dome forming, identifying an optimal clamping force that suppresses prepreg buckling without inducing metal cracking, which is an optimal parameter range unreported in earlier studies focused on flat or single-curved parts.

A third unresolved gap in prior research is the lack of validated non-coherent deformation models for uncured 3D complex parts (e.g., double-curved domes). Most existing studies on FML deformation (e.g., [12,15,26]) focused on flat or single-curved components: Trzepieciński et al. [12] reviewed FML-forming technologies but did not integrate material constituent, fiber orientation, and the friction effects for 3D uncured forming; Larberg et al. [26] modeled the in-plane deformation of uncured prepregs but omitted metal layer plasticity and inter-layer interaction, i.e., key factors for predicting failure modes such as metal cracking and prepreg buckling in 3D parts. This study addresses this gap by designing a double-curved dome-forming system, and validating a finite element model that couples material properties, fiber orientation, clamping force, and inter-ply friction to predict non-coherent deformation and defects.

This study takes uncured metal–FRP laminates as the research object, with its core research subject being the characterization of key technological deformation mechanisms, including inter-ply friction, and the out-of-plane bending and optimization of forming process parameters. The research purpose is to use tailored experimental methods like double-lap sliding, clamped-beam bending and finite element modeling (FEM) to reveal deformation mechanisms, thereby establishing a process optimization framework for defect-free industrial forming. This work helps establish an experimental–numerical framework that integrates deformation mechanisms and optimized parameters, fulfilling the needs for integrated deformation control in complex metal–FRP laminate manufacturing.

## 2. Materials and Methods

### 2.1. Materials

The raw materials used for the metal–FRP laminates were metal sheets of aluminum alloy 2024-T3 and stainless steel 304L, as well as the fiber-reinforced prepregs of S2-glass/FM-94 (GFRP) and T300-carbon/MTC510 (CFRP) (SHD Composites, Sleaford, UK). The surface of metal sheets was pre-treated by anodizing and acetone washing procedure with a single layer of 0.5 mm thickness [1,2]. The metal composite materials consist of two layers of metal sheet and one layer of fiber prepregs which are known as a 2/1 layup. Each fiber layer includes two cross-plied unidirectional (UD) prepregs with a total thickness of 0.3 mm which is a methodical parameter selected for experimental sample manufacturability but consistent with industrial aerospace specifications [5,10]. This thickness balances sufficient intra-ply shear deformation [25] and structural stability to avoid premature prepreg tearing, which is particularly relevant for industrial components like wing ribs where FRP thickness directly affects deformation coordination with metal layers [10]. The UD fiber ply-oriented at 0° corresponds with the rolling direction of metal sheet. The mechanical properties of the material constituents in hybrid laminates are shown in Table 1, and the behavior of the metal sheet cannot be affected under the studied temperature ranges. Two parameters of elastic modulus and ultimate strength for the UD-reinforced fiber prepregs represent the properties in the fiber orientation of 0° and 90°, respectively.

### 2.2. Experimental Setup

**Figure 5:**
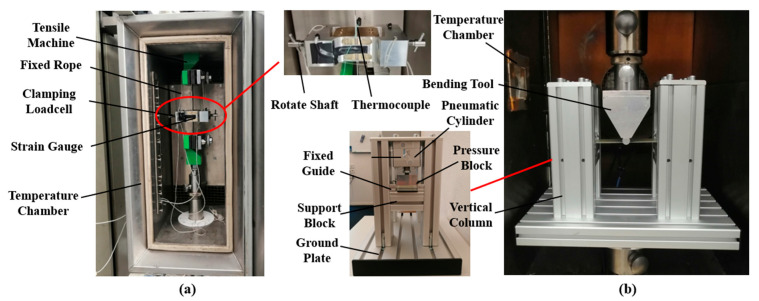
Experimental setup for the inter-ply friction and flexural bending test of uncured metal–FRP laminates [34,38]: (**a**) Double-lap sliding test setup; (**b**) Clamp-beam bending test setup.

The deformation of uncured metal–FRP laminates involves multiple interacting mechanisms, including the inter-ply sliding at metal–prepreg interfaces, the flexural bending of the entire stack, and in-plane plastic deformation of metal layers. This study represents the first systematic investigation of inter-ply friction mechanisms in such uncured laminates. To quantify the interfacial friction behavior, double-lap sliding tests were conducted to measure coefficients of friction under varying sliding conditions [36]. The experimental setup, illustrated in Figure 5a, utilizes a Zwick 20 kN universal testing machine) (ZwickRoell, Delft, Netherlands). equipped with a temperature-controlled chamber. Samples were mounted in the machine and subjected to controlled normal loads via the load cell. Additionally, the flexural bending characteristics of uncured laminates were evaluated using a custom clamp-beam bending test configuration [39]. As shown in Figure 5b, the bending apparatus features two pneumatic cylinders mounted on the machine’s lower platen, with a bending punch aligned along the vertical centerline and attached to the upper tool for precise deformation control. The 10 mm/min sliding rate in double-lap friction tests and 5 mm/min punch rate in clamped-beam bending tests are methodical parameters chosen to ensure stable data acquisition. Importantly, these rates overlap with industrial forming rates (3–15 mm/min) for uncured Al–FRP laminates [16], where slower rates are used for complex curved parts to mitigate wrinkles and faster rates for simple shapes [16,40].

**Figure 6:**
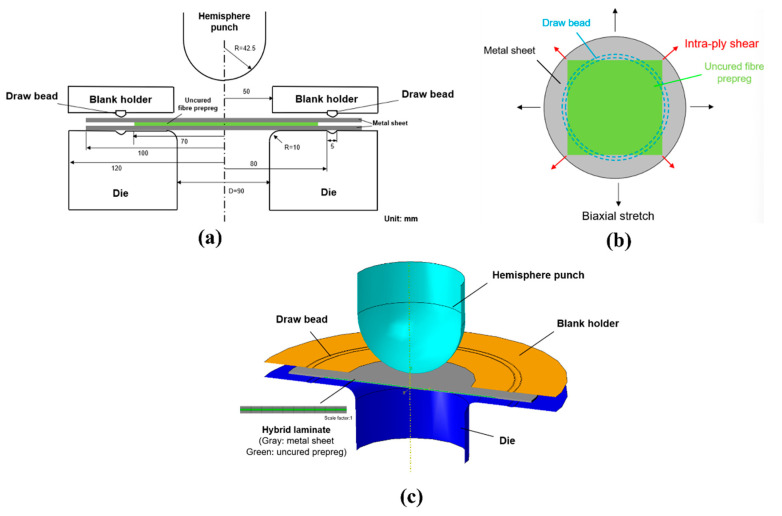
Process of the investigation of non-coherent deformation for uncured metal–FRP laminates: (**a**) Concept design; (**b**) Deformation mechanisms; (**c**) Finite element model.

To comprehensively investigate three-dimensional deformation behavior across individual layers and metal–prepreg interfaces, a specialized double-curved dome forming tool with draw bead was developed (Figure 6a). The hybrid laminate with a circle shape of 70 mm radius for metal sheet layer and a square shape of 100 × 100 mm for the fiber prepreg layer is placed symmetrically on the die surface and clamped by blank holder. As shown in the schematic graph presented in Figure 6b, the metal sheet and fiber prepreg deform independently through bias-stretch and intra-ply shear, and the different-shape design contributes to the study of a “harmony” deform for the individual layers. Furthermore, the concept of draw bead with a depth of 1 mm and length of 5 mm is proposed to constrain the material flow, as well as achieve a part without cracks and wrinkles. The dome punch with a radius of 42.5 mm moves vertically down the centerline of the laminate to a special displacement. In addition to the different material constituents studied in the research, the effects of clamping force and intra-ply friction were compared as well according to the bending and friction tests. Table 2 presents the test parameters used for the forming simulation, and the model was conducted varying one parameter at a time while keeping the other parameters at the baseline value. To simplify the temperature condition, the friction coefficient (µ) of 1 is regarded as the forming condition of room temperature (RT). Then, the frictional contacts where µ = ∞ (Tie), 0.5, 0.2 were created in the states of full cure, medium friction, and low friction, respectively.

### 2.3. Finite Element Model

The application of a finite element model (FEM) made it efficient to analyze the deformation mechanisms of the uncured hybrid laminates and provide guidance for actual experimental testing. In the research, finite element analysis, software Abaqus (version 6.14, Dassault, Paris, France) was applied to simulate the press forming process of uncured laminates by using the stress–strain distribution of the metal sheet, as well as the unique anisotropic properties of the fiber-reinforced prepreg. The non-coherent deformation model of the double-curved dome part is shown in Figure 6c. The elastic–plastic properties of the metal sheets and the elastic constants for the uncured prepreg lamina, shown in Table 1, were imported into the material property module in Abaqus. In the finite element model, all tools, including the dome punch, die, and blank holder, were modeled as discrete rigid shell elements with a mesh size of 2 mm. Metal sheets and uncured fiber-reinforced polymers were created as a four-node doubly curved conventional shell element with reduced integration (S4R). The same mesh size of 2 mm was selected as the optimal balance between accuracy and efficiency, since it avoids the numerical dispersion of 3–4 mm meshes and the unnecessary computational cost of 1 mm meshes. This assumption ensures that the predicted failure modes are consistent with experimental observations without overburdening calculations. The uncured laminate was placed symmetrically on the die surface with no initial stress or pre-deformation. Then, the laminate structure in the simulation model was represented in the composite layup module, where all layers and their parameters such as orientation, thickness, property, and relative location were defined and assembled into the forming simulation. The die and blank holder fixed eliminate tool deformation as a variable, ensuring that all observed laminate deformation was attributed to material behavior rather than tool flexure. The dome punch was controlled via displacement with a constant downward velocity of 5 mm/min, and displacement control was selected to ensure consistent forming depth across simulations. The tool–laminate contact adopted a penalty friction formulation with a constant friction coefficient (μ = 0.12), which selected for its computational efficiency in large-deformation problems and its alignment with experimental measurements of tool–laminate friction. A surface-based friction contact formulation incorporating both elastic and viscous contributions was used, with friction coefficients varying as shown in Table 2. This formulation was chosen to capture the dynamic inter-ply sliding behavior of uncured laminate, where resin viscosity and surface asperity contact jointly govern friction.

## 3. Results and Discussion

### 3.1. Characterization of Inter-Ply Friction Model

#### 3.1.1. Numerical Implementation of Experimental Results

**Figure 7:**
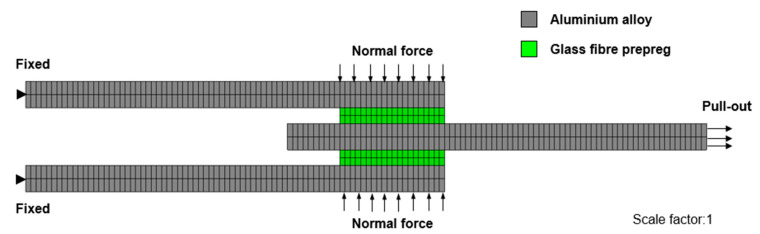
Numerical simulation for the double-lap sliding test of uncured metal–FRP laminates.

**Figure 8:**
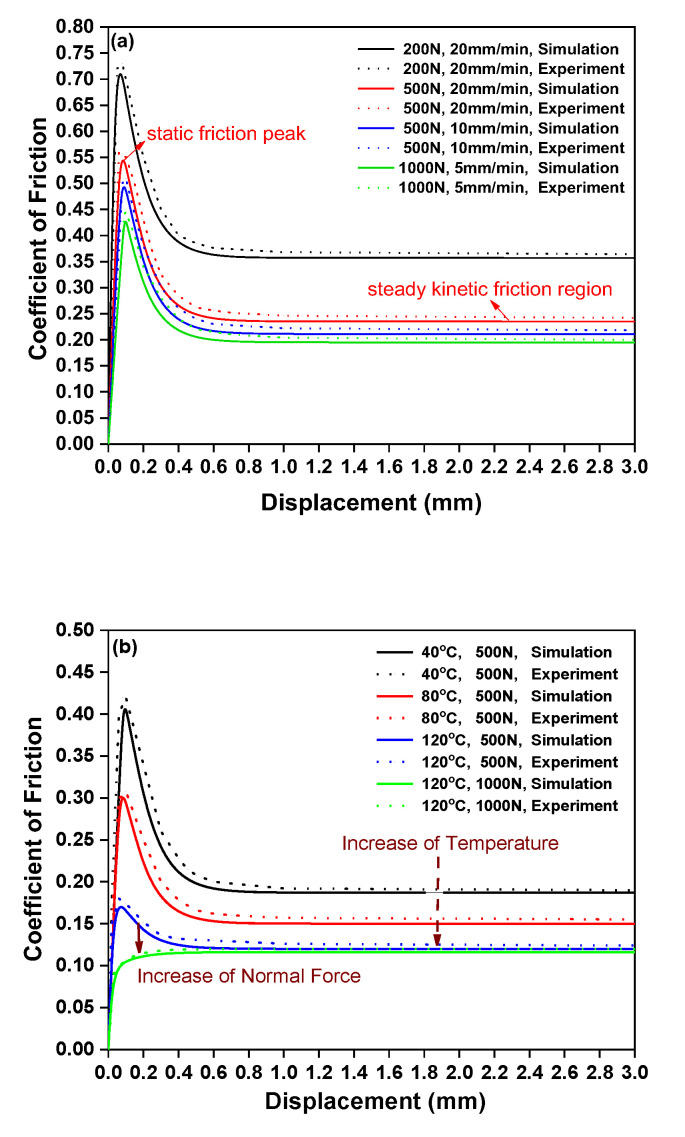
Comparison of numerical and experimental results for inter-ply friction of metal–FRP laminates: (**a**) room temperature 23 °C; (**b**) sliding rate 10 mm/min.

Inter-ply friction behavior was investigated through numerical and experimental analysis (Figure 7). As shown in Figure 8, the numerical model effectively captured two distinct friction states: (1) a peak state (static friction) and (2) a steady sliding state (kinetic friction). The results show that the friction coefficient increases with sliding rate; this points to a Newtonian shear-dominated mechanism, where epoxy matrix shear stress rises with shear rate. Conversely, friction decreased with increasing normal force. At low normal forces, the interface between the fiber-reinforced prepreg and the aluminum sheet exhibited rough surface contact with prominent asperities, leading to higher friction. However, under higher normal forces, resin percolation occurred, forming a more uniform lubrication film that reduces friction coefficients. Additionally, Figure 8b demonstrates that both static and kinetic friction coefficients decline as temperature increases from 40 °C to 120 °C. This trend is attributed to reduced resin viscosity and enhanced lubrication due to thermal softening and resin flow, as detailed in reference [36].

While the numerical model provides a reliable prediction of inter-ply friction under varying sliding rates, normal forces, and temperatures, minor discrepancies exist between simulation and experimental results. Specifically, the model slightly overestimated the static friction coefficient and onset sliding displacement. Additionally, it predicted a steady kinetic friction state earlier than observed experimentally, where the friction coefficient continued to decrease gradually. These discrepancies arise from three key physical factors not fully captured in the current model, each tied to the unique behavior of uncured prepregs. Firstly, the numerical model assumes that resin flows instantaneously to fill surface asperities under normal force, leading to a uniform reduction in interfacial roughness. However, in experiments, the epoxy resin exhibited a time-dependent viscoelastic flow [36]. Secondly, the model defined the onset of kinetic friction as the point where interfacial shear stress exceeds the static friction threshold, assuming no further change in surface topography. In experiments, however, initial sliding induced mechanical polishing of the metal–prepreg interface: asperity tips on the anodized Al surface were worn down by fiber bundles in the prepreg, and resin was squeezed into the newly formed micro-grooves [31]. Thirdly, the numerical model assumed a constant kinetic friction coefficient, implying a stable lubrication film thickness once sliding began. In experiments, continuous sliding promoted further resin migration to the interface, increasing the lubrication film thickness over time [33].

Despite these discrepancies, they were negligible considering experimental uncertainties and do not compromise the model’s validity. The physical mechanisms discussed above further confirm that the static–kinetic friction model captured the core drivers of uncured metal–prepreg interfacial behavior, while the discrepancies highlighted opportunities to refine the model incorporating the time-dependent resin flow in future work.

#### 3.1.2. Comparison with Other Numerical Models

**Figure 9:**
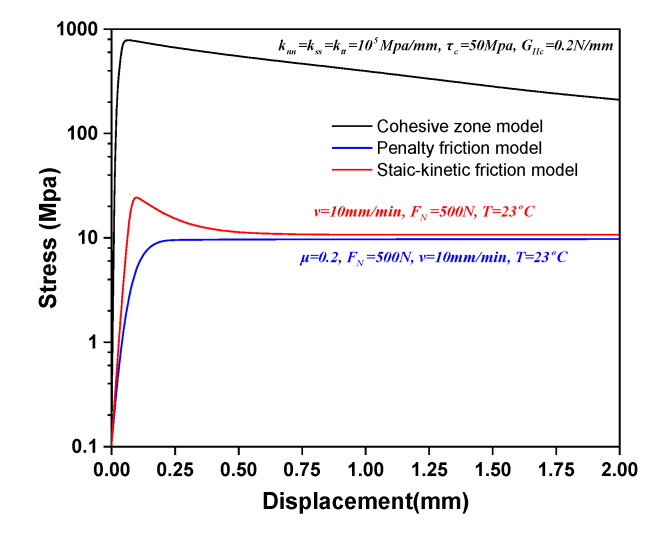
Comparison of three numerical models at the metal–prepreg interface of metal–FRP laminates.

**Figure 10:**
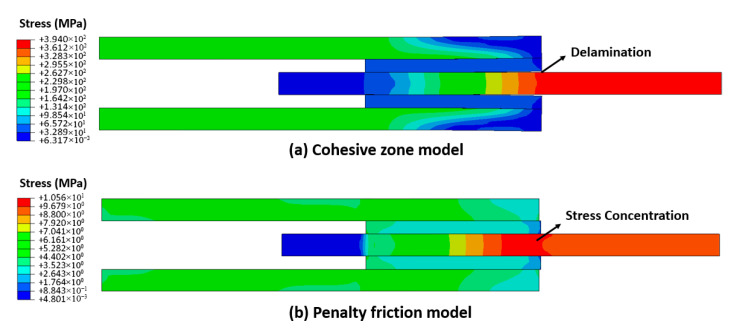
Numerical results of two different contact models at the displacement of 1 mm of metal–FRP laminates: (**a**) Cohesive one model; (**b**) Penalty friction model.

To further validate the accuracy of the static–kinetic friction model, two alternative numerical approaches—the penalty friction model and the cohesive zone model—were employed to simulate inter-ply contact behavior. Figure 9 compares their stress–displacement responses under identical loading conditions (normal force: 500 N). The cohesive zone model (shear stiffness: 105 MPa/mm, maximum shear strength: 50 MPa, fracture toughness: 0.2 N/mm) exhibited fundamentally different behavior from the other models. Its stress magnitude during sliding diverged by orders of magnitude, with peak stress occurring at significantly smaller displacements (Figure 9). This discrepancy arises because the model effectively imposes a tie constraint where initial sliding induces abrupt interfacial separation or fracture, and generates unrealistically high shear stresses. As evidenced in Figure 10a, this mechanism triggers premature delamination at metal–prepreg interfaces, mimicking the brittle failure of cured composites rather than the progressive deformation of uncured laminates. Consequently, the cohesive zone model proved unsuitable for simulating inter-ply contact across diverse press forming conditions. In contrast, the penalty friction model (constant μ = 0.2) matched the static–kinetic model’s steady-state sliding response despite stress concentration at initial sliding zones (Figure 10b). However, it failed to replicate the static friction peak observed experimentally, particularly under high-temperature conditions where resin viscosity reduction promotes smoother transitions to kinetic friction. This limitation renders it inadequate for comprehensive forming simulations. Through systematic comparison, the static–kinetic friction model emerged as the most accurate representation of inter-ply friction in fiber–metal laminates, effectively capturing rate-dependent, normal force-sensitive, and temperature-driven frictional behavior essential for a realistic forming process.

### 3.2. Characterization of Flexural Bending Model

#### 3.2.1. Load and Displacement Response

**Figure 11:**
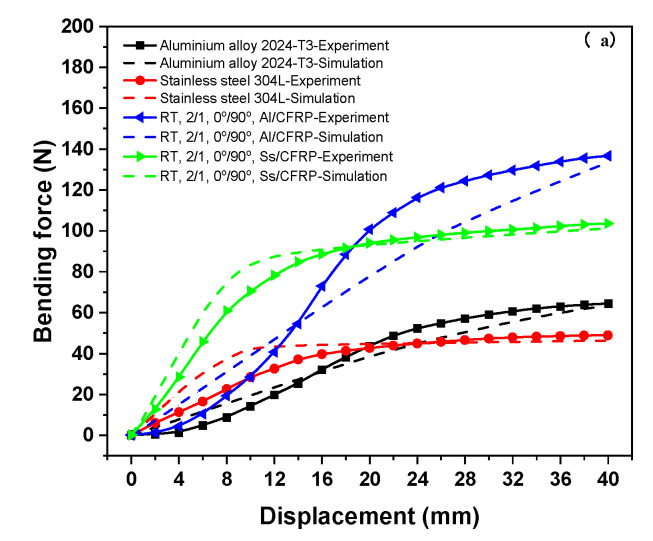

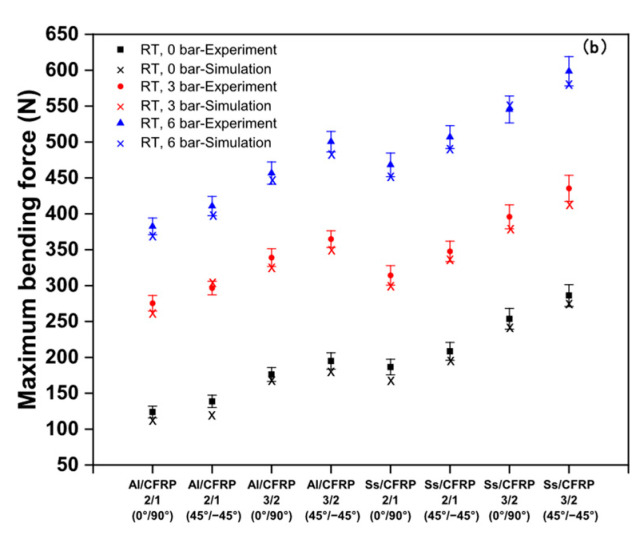
Experimental and numerical results on the bending force response of the test materials: (**a**) room temperature (RT) with the clamping pressure of 0 bar; (**b**) room temperature (RT) with various clamping pressures.

In the clamped-beam bending process, the load–displacement response serves as a key quantitative indicator for characterizing the flexural behavior of the test materials. Figure 11a shows a comparative analysis of experimental and finite element simulation results for the bending load–displacement response under unclamped conditions at room temperature. During the initial bending phase, the simulated bending force exhibited a slightly steeper increase compared to experimental measurements. This discrepancy primarily arises from the delayed response of the test machine during the initial punch–specimen contact. However, as punch displacement increases, the bending forces gradually converge, reaching alignment at 40 mm displacement. The close agreement in maximum bending force values validates both the experimental methodology and finite element model, particularly in the final stages of bending. Regarding material constituent effects, the maximum bending force for single-layer Al2024-T3 and Al/CFRP laminates significantly exceeded that of the single-layer Ss304L and corresponding Ss/CFRP laminates under identical test configurations. Additionally, stainless steel-based materials demonstrate earlier force stabilization during bending, which correlates with the stress–strain characteristics that the higher yield stress of the aluminum alloy generates greater punch reaction forces, while the lower yield stress of the stainless steel promotes earlier plastic deformation, resulting in slower stress and bending force development.

The application of clamping pressure is one of the novelties in the research, and the investigation of clamping effects on the flexural behavior has become significant. Therefore, Figure 11b presents the test and simulation results of the maximum bending force for various structures of Al/CFRP and Ss/CFRP laminates under three clamping pressures at room temperature. It is obvious that the increase in clamping pressure tended to increase the maximum bending force at the end of the bending test for all the test configurations. One possible explanation is that increased clamping pressure restricts material flow along the longitudinal direction, thereby requiring greater bending force to achieve equivalent deformation. This phenomenon can also be understood in terms of plastic strain development, where higher clamping pressure results in greater plastic strain. Consequently, the material requires higher stress to induce plastic deformation, which begins earlier in the process under these conditions. Moreover, the increasing of laminate layup from a 2/1 to a 3/2 hybrid structure further contributed to the rise in bending force. The additional layers played a dominant role in enhancing bending resistance, which aligns with findings from prior studies on three-point bending and fixed-beam bending tests [39,40]. Also, the 45°/−45° fiber-oriented hybrid laminates exhibited marginally lower bending forces than their 0°/90° counterparts under identical conditions. This reduction can be attributed to intra-ply shear effects at maximum displacement.

#### 3.2.2. Bending Characterization of Spring-Back

**Figure 12:**
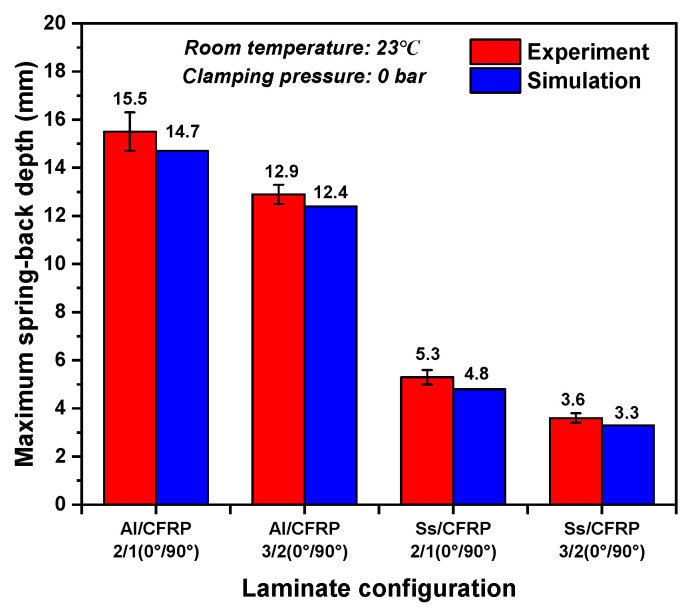
Experimental and simulation results for the spring-back depth for various laminate configurations.

**Figure 13:**
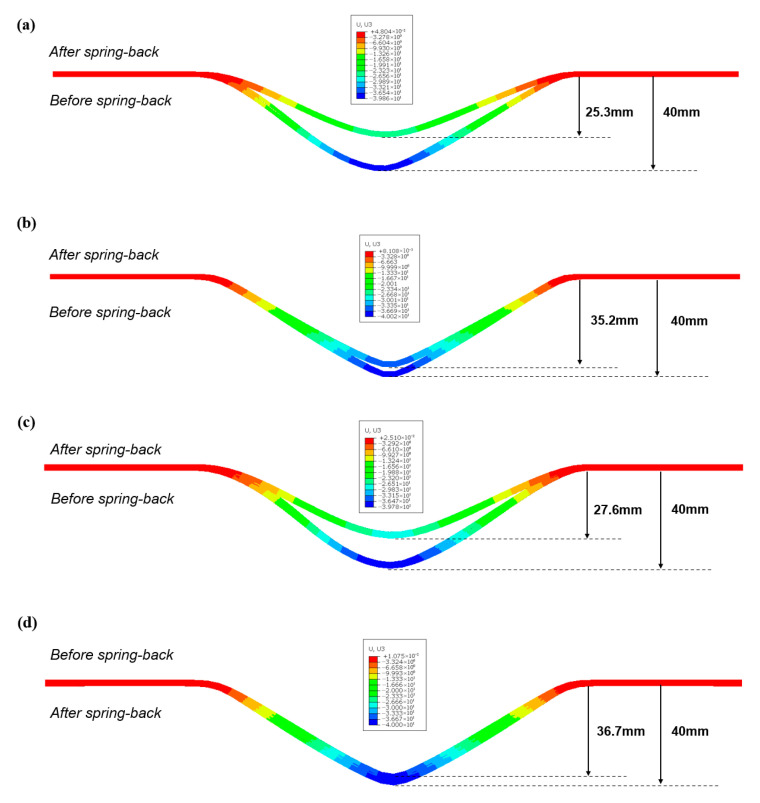
Numerical results of the vertical displacement (U3) before and after spring-back for different materials under the conditions of zero clamping pressure and room temperature (23 °C): (**a**) 2/1, 0°/90°, Al/CFRP; (**b**) 2/1, 0°/90°, Ss/CFRP; (**c**) 3/2, 0°/90°, Al/CFRP; (**d**) 3/2, 0°/90°, Ss/CFRP.

The spring-back evolution following clamped beam bending provides valuable insight into the flexural behavior of laminates. Both experimental and numerical methods were employed to analyze the deformation differences before and after spring-back. Figure 12 presents the spring-back depths for various laminate configurations at room temperature without clamping pressure. Notably, the influence of fiber orientation and processing temperature on uncured metal–CFRP laminates was excluded, as these effects were deemed negligible. The results indicate that the aluminum-based laminates exhibited greater spring-back than their stainless steel-based counterparts, while increased laminate layup reduced spring-back depth. This trend aligns with the stress–strain relationship, whereby a higher elastic modulus and lower yield strength of the stainless steel leads to a reduced elastic response compared to aluminum alloys at equivalent deformation levels. Additionally, the enhanced layup significantly mitigated spring-back due to the larger bending radius of the outer-metal sheet layers of uncured laminates. This geometry altered strain distribution and elastic recovery, as supported by prior studies [39]. Moreover, Figure 13 illustrates the numerical color maps of vertical displacement (U3) before and after spring-back for various laminate configurations under identical conditions. It was observed that the vertical displacement and shapes after unloading for aluminum-based and stainless steel-based hybrid laminates were different. The increase in laminate layup and bending radius gradually hinders the vertical movement, and the materials become flat on the flange regions.

### 3.3. Characterization of the Non-Coherent Deformation Model

#### 3.3.1. Effect of the Material Constituent

**Figure 14:**
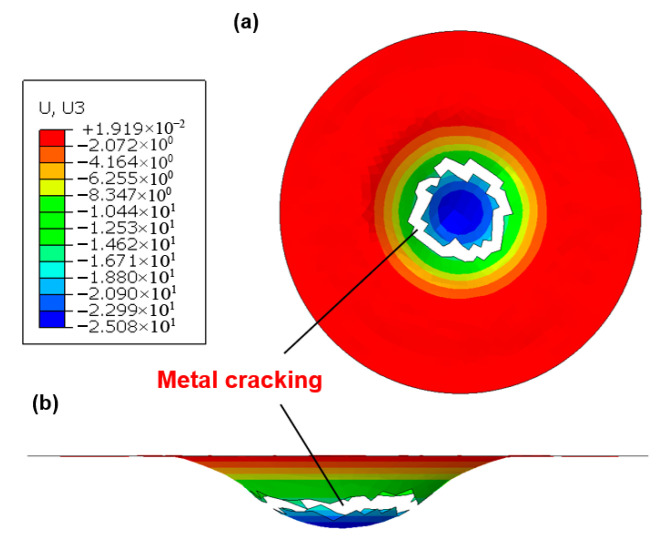
Numerical simulation of vertical displacement (U3) for Al/GFRP laminates at the maximum value before failure. Test conditions: room temperature (23 °C), zero clamping force. (**a**) XY-plane view of the aluminum layer; (**b**) XZ-plane view of the aluminum layer.

**Figure 15:**
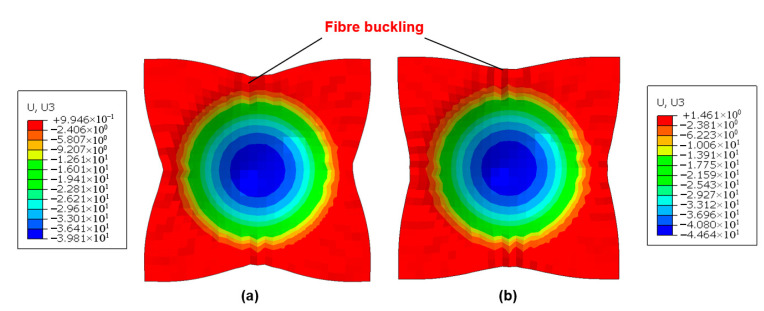
Numerical simulation of vertical displacement (U3) for stainless steel (Ss)-based hybrid laminates at the maximum value before failure. Test conditions: room temperature (23 °C), zero clamping force. (**a**) Ss/CFRP laminate; (**b**) Ss/GFRP laminate.

During laminate press forming, the metallic and composite constituents significantly influence the deformability and failure mechanisms of hybrid materials. Figure 14 presents the numerical simulation results of vertical displacement (U3) prior to failure for an Al/GFRP laminate under room temperature and zero clamping force conditions. The primary failure mode observed was metal cracking, which is numerically represented through element deletion once forming limits are exceeded. As illustrated, the lower aluminum sheet initiated cracking at a displacement of 25.08 mm, with failure predominantly localized in the central region of the metal layer. In contrast, the GFRP interlayer, though not explicitly depicted in the figure, remained intact and maintained contact with the outer metal layers at this displacement. Furthermore, the fiber prepreg type (GFRP vs. CFRP) exhibited negligible influence on metal cracking behavior and maximum forming depth in aluminum-based laminates. This is attributed to the comparatively lower failure strain of aluminum alloys relative to stainless steel, while the fiber prepreg experiences neither significant intra-ply shear strains nor failure at equivalent displacements. Conversely, substituting aluminum alloys with stainless steel drastically alters the failure mode. In stainless steel-based laminates (e.g., Ss/CFRP and Ss/GFRP), prepreg buckling becomes the dominant failure mechanism, while the outer metal layers remain crack-free (Figure 15). Under identical forming conditions, the critical vertical displacements (U3) before failure were 39.81 mm for SS/CFRP and 44.64 mm for Ss/GFRP, respectively. Prepreg buckling arises from layer overlap induced by intra-ply shear strains surpassing the locking angle. Notably, GFRP demonstrated a higher locking strain than CFRP, resulting in greater formability before failure.

#### 3.3.2. Effect of Clamping Force

**Figure 16:**
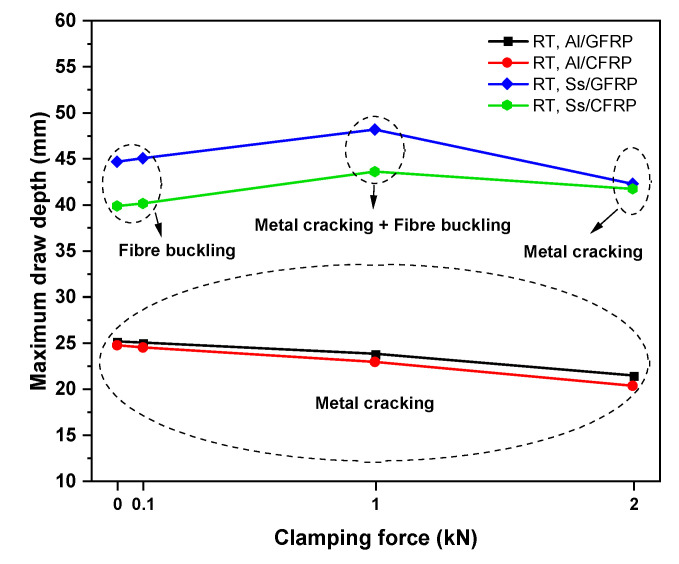
Numerical simulation of maximum draw depths and corresponding failure modes for metal-FRP laminates. Test conditions: room temperature (23 °C), clamping forces of 0 kN, 0.1 kN, 1 kN, and 2 kN.

Figure 16 illustrates the simulation results regarding the maximum draw depth and its corresponding failure mode under four clamping force conditions at room temperature. For aluminum-based hybrid laminates incorporating both CFRP and GFRP, increasing the clamping force tended to reduce the maximum draw depth, though the effect was modest. This is primarily because elevated biaxial stress induces earlier cracking of the aluminum sheet. The primary failure mode for these laminates was metal cracking, with no observed buckling in the prepreg layers. However, a larger draw depth under lower clamping force did not indicate better formability for Al/CFRP and Al/GFRP laminates, as metal wrinkling occurred in flange regions. For stainless steel (Ss)-based hybrid laminates, the failure mode varied complexly under different clamping forces. Increasing the clamping force from 0 kN to 1 kN increased the maximum draw depth, with failure characterized by both metal cracking and prepreg buckling. When the clamping force reached 2 kN, metal cracking became the dominant failure mode with a reduced failure depth, and prepreg buckling no longer occurred. This suggests that increasing the clamping force delays prepreg buckling.

**Figure 17:**
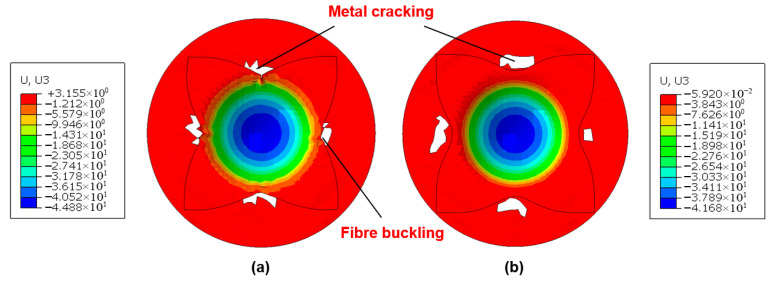
Numerical simulation of vertical displacement (U3) for Ss/CFRP laminates at the maximum value before failure. Test conditions: room temperature (23 °C), and clamping forces of (**a**) 1 kN and (**b**) 2 kN.

**Figure 18:**
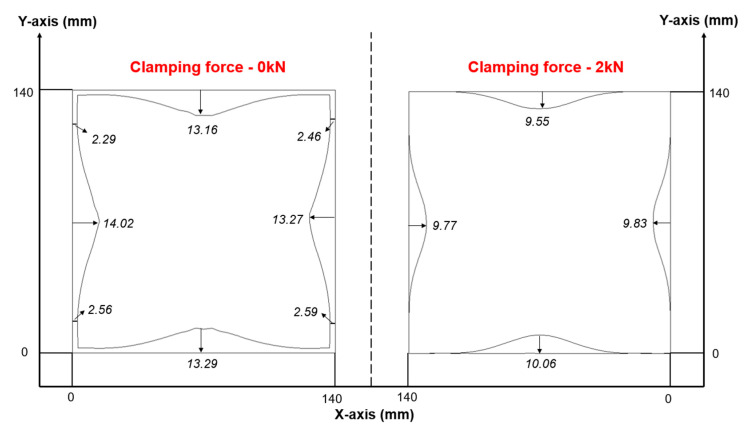
Comparison of initial and final CFRP prepreg shapes for Ss/CFRP laminates from numerical simulation. Test conditions: room temperature (23 °C), draw depth of 40 mm, and clamping forces of 0 kN and 2 kN.

A more detailed illustration of the distinct failure modes for SS/CFRP laminates under 1 kN and 2 kN clamping forces is provided in Figure 17. Notably, a higher clamping force reduced the maximum failure depth, with metal cracking occurring in the inner radius regions. Firm clamping at the four-square corners of the CFRP layer reduced prepreg buckling by inducing tensile forces in the prepreg layers mediated by friction at the draw bead–metal and blank holder–metal interfaces. To investigate friction effects, Figure 18 compares the initial and final prepreg shapes of the Ss/CFRP laminates under two clamping forces at room temperature and a draw depth of 40 mm. At a clamping force of 0 kN, the CFRP layer flowed more easily into the central region, increasing the likelihood of prepreg buckling. However, at 2 kN, the four-square corners of the CFRP layer exhibited almost no movement. Since clamping force is only applied to these four-square corners, its impact on the deformability of the 0°/90° CFRP in the four central edge regions is limited, as measured relative displacements are nearly identical. Thus, a clamping force of 1 kN can be considered an optimized parameter for enhanced deformability which has direct practical significance for aerospace applications.

#### 3.3.3. Effect of Fiber Orientation

**Figure 19:**
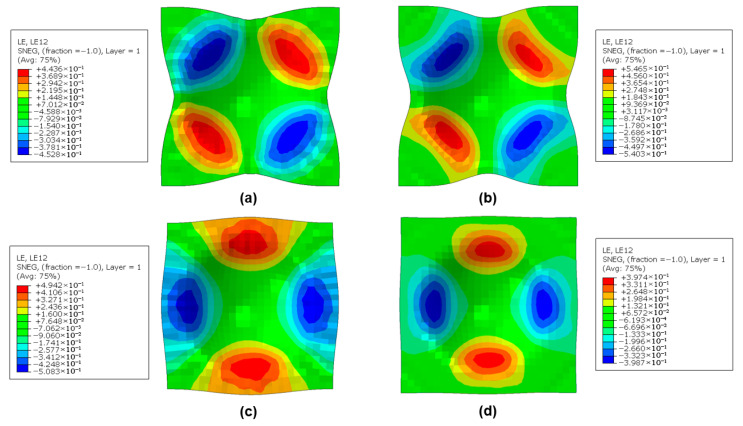
Numerical comparison of shear strain distributions (LE12) in the CFRP prepreg layer of Ss/CFRP laminates. Test conditions: room temperature (23 °C), draw depth of 40 mm. (**a**) 0 kN, 0°/90°; (**b**) 2 kN, 0°/90°; (**c**) 0 kN, 45°/−45°;(**d**) 2 kN, 45°/−45°.

In order to analyze the intra-ply shear mechanism of the middle prepreg layer, the distributions of shear strain under two clamping forces, as well as two fiber orientation conditions were evaluated at a draw depth of 40 mm at room temperature. Figure 19 shows that the deformation during forming for the cross-plied UD CFRPs which oriented at 45°/−45° and 0°/90° was different. The maximum shear strain occurred at diagonal location along the 45-degree direction for the 0°/90° layups, while the 45°/−45° layups underwent the maximum shear strain at a central area along the horizontal and vertical direction. With the increase in clamping force, the evolution of shear strain seemed to have an opposite trend. Although the increasing clamping contributed to the deformation of both layups, the CFRP with 0°/90° layup can exert its deformability in a better mechanism. As the length and width increased at the maximum shear strain regions for 45°/−45° layup, the compression force it induces tended to have the failure of prepreg buckling. Therefore, the CFRP material which was oriented at 45°/−45° was not suitable for forming the dome part under the same condition as prepreg requires intra-ply shear for deformation.

#### 3.3.4. Effect of Inter-Ply Friction

**Figure 20:**
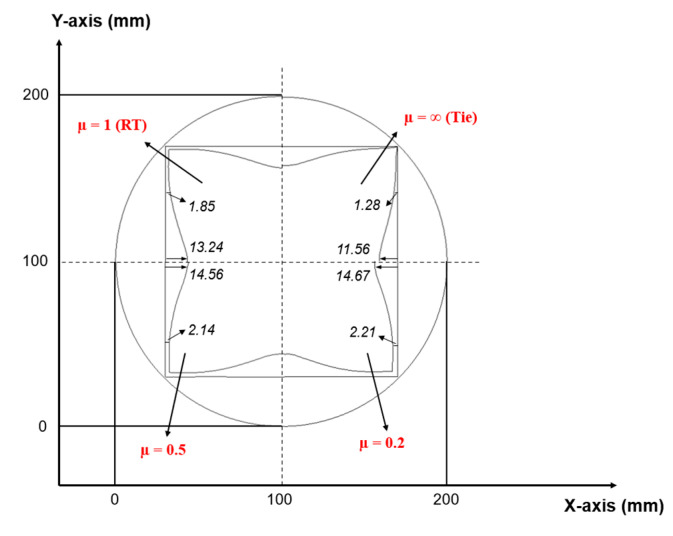
Comparison of initial and final laminate shapes for Ss/CFRP laminates from numerical simulation. Test conditions: room temperature (23 °C), clamping force of 1 kN, draw depth of 40 mm, and inter-ply friction coefficients (μ) of ∞ (tie), 1 (RT), 0.5, and 0.2.

**Figure 21:**
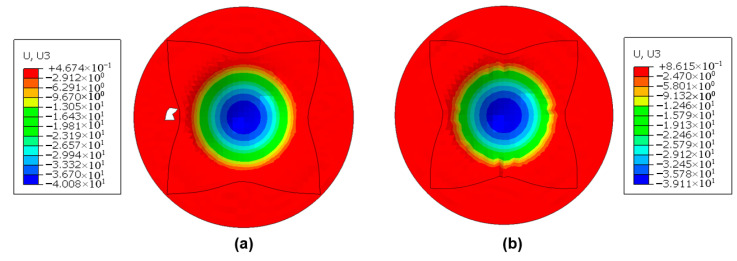
Numerical simulation of vertical displacement (U3) for Ss/CFRP laminates before failure. Test conditions: room temperature (23 °C), clamping force of 1 kN, and inter-ply friction coefficients (μ) of (**a**) ∞ (tie) and (**b**) 0.2.

The significance of inter-ply friction can be demonstrated by comparing simulations with a fully constrained “tie” condition (µ = ∞) and cases with varying frictional coefficients. Figure 20 illustrates the laminate shapes before and after forming at a draw depth of 40 mm for Ss/CFRP laminates, as obtained from numerical simulations. The influence of friction on inter-ply sliding was evident from the displacement of the edge for the prepreg layer. When the metal–prepreg interface was fully constrained simulated as a cured laminate, the CFRP layer exhibited a maximum horizontal displacement of 11.56 mm, with the minimal gap formation at the center. As the inter-ply friction decreased, both the horizontal displacement and the central gap increased significantly. This indicates that higher friction restricted inter-ply sliding, thereby constraining deformation and delaying CFRP buckling. Furthermore, the choice of friction coefficient distinctly affected failure modes. Figure 21 presents numerical results at a maximum vertical displacement for the Ss/CFRP laminates under 1 kN clamping force. Under tie constraints, failure is characterized by metal cracking at greater depths, whereas a lower friction coefficient (µ = 0.2) results in the dominant prepreg buckling. These findings suggest that optimizing inter-ply friction can enhance the formability of uncured laminates by mitigating both metal cracking and prepreg buckling failures.

#### 3.3.5. Discussion of Other Effects

Apart from the above-mentioned effects on the numerical research for the press forming of uncured metal–FRP laminates, multiple other influencing factors need to be considered. A few of them will be discussed briefly in this section. First, the application of a draw bead aims to control the material flow of both metal sheets and prepreg layers by applying a dedicated clamping force. Therefore, the size and location of the draw bead are critical in such research. Since the ring-shaped draw bead covers all sections of the circular metal blanks, the larger bead or a higher blank holder pressure result in more restriction of the metal flow, which cause earlier cracking during forming. However, in these cases, the defect of metal wrinkling is postponed if the draw bead and blank holder are properly applied. For the effects of the draw bead on the uncured prepreg, it is obtained that the larger the size on four-square corner regions that is clamped, the more pronounced the intra-ply shear of the prepreg layers, which is beneficial for prepreg deformation. The same effect can be achieved by increasing the pressure.

Second, the blank diameter and thickness for the metal sheet affect the laminate deformability as well, which is proven by studies on the deep drawing process [11,16]. From those studies, it is clear that the blank diameter has an optimum for a given material and thickness. Increasing the diameter shifts the balance between drawing and stretching toward stretching, while decreasing the diameter shifts the balance toward drawing. A decreasing thickness results in more primary and secondary wrinkling problems. For the uncured prepreg, the size effects are less dominant.

Third, another parameter that affects the results is the friction between the layers during forming. An increased temperature lowers the resin viscosity and friction coefficient at the metal–prepreg interfaces, while the increase in fiber shear angles as the temperature increases for cross-plied UD prepregs [39] will further accelerate the occurrence of prepreg buckling. Therefore, the proper heating of the uncured laminates under a medium temperature and friction condition is beneficial to form a high-quality dome part without the failure of metal cracking and prepreg buckling at the same time. In this respect, the type of resin and the fiber architecture also play their roles.

The last factor to be mentioned is the product shape. In this numerical study, only a circular blank was investigated to be deformed in a dome shape. When other shapes, e.g., box shapes, are investigated, the strategy to induce the two deformation mechanisms (biaxial stretching for metals, intra-ply shear for prepregs) requires new blank designs for both metal sheets and prepreg layers. Additionally, the tool design, including the position of draw beads and size and shape of blank holder, needs to be considered. All these considerations are recommended to be explored in future research.

## 4. Conclusions

Deformation behaviors, including inter-ply friction, out-of-plane bending and in-plane plastic deformation of the uncured metal–FRP hybrid laminates, were experimentally and numerically characterized. A double-lap sliding test and a clamped-beam bending test method were designed to quantify and optimize critical process parameters for simulating non-coherent deformation under varying conditions, such as material constituent, clamping force, fiber orientation, and inter-ply friction. Key findings are summarized as follows:

(1) A static–kinetic friction model of uncured metal–FRP hybrid laminates was developed and validated using a novel friction test apparatus. The model accurately predicted the initial transient response and the transition from static to kinetic friction under different sliding rates, temperatures, and normal forces. Numerical predictions of friction coefficient–displacement trends showed excellent agreement with experimental results. For aerospace manufacturing, this model provides a quantitative tool to predict the interfacial behavior of uncured Al 2024-T3/CFRP laminates under real forming conditions, reducing wrinkle-related scrap.
(2) A flexural bending model of uncured metal–FRP hybrid laminates was established and validated via clamped-beam bending tests. The application of clamping pressure had a significant impact on the flexural behavior of the bent laminates. Higher plastic deformation in the metal layers resulted in a significant increase in bending force and a reduction in the spring-back depth after unloading, directly addressing the need for aerospace manufacturers to minimize post-processing time.
(3) The failure mode of the uncured metal–FRP hybrid laminates primarily depends on the metal constituent, with fiber-reinforced prepreg playing a limited role. The increase in clamping force contributes to the deformation of the fiber-reinforced prepreg and decreases the risk of prepreg buckling, while excessive biaxial stress and restricted material flow induce metal cracking in stainless steel-based laminates. For aerospace applications, this identifies optimal parameters: Al-based laminates require 0.1–0.5 kN clamping force to avoid cracking, while stainless steel-based laminates perform best at 1 kN.

## 5. Limitations and Future Work

While this study provides a foundational experimental–numerical framework for uncured metal–FRP laminate forming, it has two key limitations that warrant consideration. First, the non-coherent deformation analysis was restricted to double-curved dome geometries. This simplifies the boundary conditions compared to more complex aerospace component, where material flow and inter-layer interaction are more heterogeneous. The current model may not fully capture the multi-directional stress states and localized defect formation like corner wrinkling in such shapes. Moreover, the inter-ply friction model adopted simplified thermal assumptions: while temperature effects on friction coefficient were characterized, the model does not account for coupled thermal–mechanical effects such as transient temperature gradients during hot pressing or resin curing shrinkage-induced stress evolution. In industrial aerospace forming, these coupled effects can alter interfacial friction and metal plasticity, leading to deviations between simulation and real-world performance.

To address these limitations, future work will focus in three directions: (1) extending the non-coherent deformation model to complex 3D geometries (e.g., box-shaped parts) by optimizing the blank design (e.g., tailored metal/FRP blank sizes) and tooling (e.g., variable draw bead positions), as well as integrating multi-scale stress analysis to capture corner defects; (2) developing a coupled thermal–mechanical friction model that incorporates transient temperature fields (from hot press heating cycles) and resin curing kinetics (e.g., degree of cure-dependent viscosity), using in situ thermocouple measurements to validate temperature–stress–friction relationships; (3) and explore the applicability of the framework to thermoplastic-based metal–FRP laminates (e.g., Ti/PEEK systems), which are increasingly being used in high-temperature aerospace components but exhibit different deformation mechanisms (e.g., melt flow of thermoplastics) compared to the thermoset prepregs (S2-glass/FM-94, T300-carbon/MTC510) studied here.

## Figures and Tables

**Table 1:** Mechanical properties of the material constituents in metal–FRP laminates.

	Density (g/cm^3^)	Elastic Modulus (GPa)	Poisson’s Ratio	Shear Modulus (GPa)	Yield Strength (MPa)	Ultimate Strength (MPa)	Shear Strength (MPa)	Elongation at Break (%)
Aluminum alloy 2023-T3	2.7	71	0.33	28	320	480	283	16.2
Stainless steel 304L	8.0	200	0.30	77	210	574	378	45.6
UD glass fiber prepreg-FM94	2.6	54.0/9.4	0.33	5.5/2.6	-	1870/50	38.5	3.8
UD carbon fiber prepreg-MTC510	1.5	119.3/8.2	0.34	3.6/2.0	-	2282/54	99	1.3

**Table 2:** Test parameters used for the press forming process of metal–FRP laminates.

Test Parameter	Baseline Value	Additional Values Investigated
Clamping force (kN)	0	0.1, 1, 2
Fiber orientation (°)	0/90	−45/45
Inter-ply friction (µ)	1 (RT)	∞ (Tie), 0.5, 0.2

## Data Availability

The raw data used to support the findings of this study are available from the corresponding author upon request.
